# Running shoe cushioning properties at the rearfoot and forefoot and their relationship to injury: study protocol for a randomised controlled trial on leisure-time runners

**DOI:** 10.1136/bmjsem-2024-002217

**Published:** 2024-10-11

**Authors:** Laurent Malisoux, Axel Urhausen, Nicolas Flores, Daniel Theisen, Cédric Morio

**Affiliations:** 1 Department of Precision Health, Luxembourg Institute of Health, Strassen, Luxembourg; 2 Sports Clinic, Centre Hospitalier de Luxembourg Clinique d'Eich, Luxembourg, Luxembourg; 3 Luxembourg Institute of Research in Orthopedics, Sports Medicine and Science, Luxembourg, Luxembourg; 4 Decathlon SportsLab Research and Development, Lille, France; 5 ALAN – Maladies Rares Luxembourg, Luxembourg, Luxembourg

**Keywords:** Epidemiology, Intervention, Prevention, Sporting injuries, Running shoes

## Abstract

Previous work has demonstrated the protective effect of shoe cushioning on injury risk in leisure-time runners, but most models currently available on the market have greater cushioning than those investigated so far. Also, the optimal level of cushioning and the role of cushioning on the forepart of the shoe for injury prevention are still unknown. The main aim of this study is to determine whether (1) current ‘extra soft’ cushioning material at the rear part of the shoe reduces injury risk compared with stiffer material and (2) cushioning under the forepart of the shoe also contributes to injury risk reduction. This randomised trial with a 6-month intervention will involve 1000+ healthy leisure-time runners who will randomly receive one of the three running shoe versions. Study shoe versions will differ in their cushioning properties (ie, stiffness) at the rear or the forepart. Participants will self-report any lower limb or lower back problems on a dedicated electronic system every week, while the system will collect training data from the participant’s sports watch. Time-to-event analyses will be used to compare injury risk between the three study groups and to investigate the association between the runner’s characteristics, cushioning level and position, training and injury risk. The study was approved by the National Ethics Committee for Research (Ref: 202405/02 v2.0), and the protocol has been registered on https://clinicaltrials.gov/ (NCT06384872, 02/08/2024). Outcomes will be disseminated through presentations at international conferences and publications in peer-reviewed journals, popular magazines and specialised websites.

**WHAT IS ALREADY KNOWN ON THIS TOPIC**

**WHAT THIS STUDY ADDS**

- This is the first randomised trial to investigate the effect of ‘extra soft’ cushioned shoes (about 40 N/mm) and cushioning location (ie, rear and forepart of the shoes, respectively) on running-related injury risk.

**HOW THIS STUDY MIGHT AFFECT RESEARCH, PRACTICE OR POLICY**

- The study outcomes will help better understand the relationship between the runner’s characteristics, cushioning level and location, training and injury risk.
- The study outcomes will also help the industry make important decisions for designing future running shoe models.

## Introduction

 Regular physical activity prevents chronic diseases and reduces all-cause mortality.^1^ In particular, vigorous-intensity aerobic physical activity has been shown to provide greater benefits for the risk of premature mortality.^2^ However, physical activity has declined globally for decades, largely driven by occupational, domestic and commuting physical activity reductions.^3^ The negative impact of an inactive lifestyle on health is one of the main motivations for adults to perform more physical activity during their leisure time.^4^ Long-distance running is one of the most popular physical activities across the world.^5^ Given the popularity of leisure-time running, its long-term mortality benefits,^6^ as well as the superior benefits running may confer over other types of vigorous-intensity physical activity for the prevention of chronic diseases and premature mortality,^7^ long-distance running appears to be one of the most cost-effective lifestyle interventions from a public health perspective.^8^

One of the main drawbacks of running is the high risk of sustaining a running-related injury,^9^ which is considered a major barrier to continued participation.^10^ Running-related injury incidence has remained high during the last 40 years, with an overall incidence rate of 50% per year.^11^ Most running-related injuries develop progressively, resulting from an imbalance between repetitive loading of the musculoskeletal system and tissue load capacity.^12^ More specifically, tissue microdamage resulting from repetitive loading is a stimulus for remodelling and adaptation. Yet, it may eventually lead to tissue failure (ie, tear, rupture, fracture) without appropriate repair.^13^

In relation to the repetitive loading of the musculoskeletal system, Body Mass Index has been associated with injury risk in runners.^14 15^ This observation suggests that the increased physical stress due to extra body weight affects injury risk. Surprisingly, body mass as such has hardly ever been considered as a potential risk factor for running injury.^9^ Despite the lack of evidence on the association between body mass and injury risk, it is a common belief that heavier runners should use footwear with increased shock absorption properties. Indeed, footwear cushioning as a modifiable factor has been shown to influence impact forces and loading rate during running.^16^ Nevertheless, only lighter runners benefited from greater cushioning in our previous study,^17^ which still confirms that the runner’s weight moderates the effect of shoe cushioning on injury risk.

Since repetitive loading of the musculoskeletal system has been viewed as a concern for injury risk during running, cushioning has been the most extensively investigated shoe feature. Different solutions of shoe cushioning have been developed to attenuate impact peak forces and loading rate, as these biomechanical characteristics are associated with the development of specific running injuries.^18^ The shock absorption properties of footwear mainly result from the materials used in the sole (ie, their type, density, structure and combinations thereof), as well as from the geometry of the shoe (ie, the midsole thickness and the design of inserts). Only a few prospective studies have investigated the impact of shoe cushioning on injury risk in running.^15 17 19^ One investigation compared different types of shoe insoles and found no difference in injury risk; however, the study involved Royal Air Force recruits, and the findings may not be generalised to runners.^19^ Another study with negative results compared two versions of a standard running shoe with a narrow difference in midsole hardness (15%) and limited sample size (n<250).^15^ The study limitations suggest that these findings should be interpreted cautiously, though. Our latest study on 800+ leisure-time runners provided evidence, for the first time, on the effectiveness of greater shoe cushioning (65 N/mm vs 95 N/mm) in reducing injury risk,^17^ as well as a plausible explanation for this protective effect.^20^ Nevertheless, the optimal amount of cushioning for injury prevention according to the runner’s individual characteristics remains unknown. Besides, the market has quickly evolved since the introduction of advanced footwear technology, especially regarding foam materials, which are ‘extra soft’ nowadays. The whole footwear range (from performance to cushioning and stability shoes) uses soft or extra soft foams. At the same time, it is unknown if this evolution of shoe technology impacts injury risk. Furthermore, the role of cushioning under the forepart of the shoe for injury prevention has never been investigated.

The comfort filter paradigm has previously been suggested as a new approach to reduce injury risk.^11^ Accordingly, the authors stated that a runner should select a comfortable product using his/her comfort filter. If this paradigm is valid, a relationship should exist between cushioning, comfort and injury risk, given the protective effect of greater cushioning previously reported.^17^ Furthermore, shoe cushioning has been reported to deteriorate with mileage,^21^ which may influence running biomechanics.^22^ Therefore, cushioning perception and comfort changes could inform on shoe deterioration and injury risk.

There is also a growing interest in the cumulated workload outside of scheduled training.^23 24^ Indeed, a 24-hour integrative perspective on training is potentially relevant for injury risk management, as conditions outside training significantly modulate the total workload and training adaptation and recovery. This could be even more relevant in leisure-time athletes due to a generally lower training volume and, thus, a relatively higher contribution of additional activities outside running training to tissue load. The quantification of the 24 hours weight-bearing and locomotion activities in runners may provide insight into the role of off-training behaviour on injury risk and highlight the need to control off-training activities in sports injury research. To our knowledge, no previous study has addressed this question.

## Objectives

The main purpose of this study is to investigate the effect of different cushioning solutions on injury risk in leisure-time running. The project focuses on the influence of cushioning properties at the rear and the forepart of the running shoes on musculoskeletal problems in leisure-time runners. The primary objective of this study is to determine whether ‘extra soft’ cushioning material (ie, global stiffness: 40±5 N/mm) at the rear part of the shoe reduces injury risk compared with stiffer material (ie, global stiffness: 60±5 N/mm) in leisure-time runners. The secondary objectives of this study are to investigate whether (1) cushioning under the forepart of the shoe also influences injury risk, independently of the cushioning at the rear part, (2) the effects of cushioning properties and position on injury risk depend on the runner’s body mass and (3) perception of cushioning is related to both shoe cushioning and injury risk. The present study will also explore (1) if other weight-bearing locomotion activities (ie, number of steps per day in addition to the steps from running) represent an independent risk factor for running-related injury, (2) the role of (biological) sex in injury mechanism, as previous work showed that injury risk and risk factors may be different in men and women,^25^ and its potential moderation effect, (3) how acute changes in training load affect injury risk and (4) if disparities in injury risk exist according to the socioeconomical status of the runners (eg, level of education, socioprofessional category, gross annual household income).

Our main hypothesis is that greater shock absorption properties at the rear part of standard running shoes are associated with lower injury risk in recreational runners. Our secondary hypotheses are (1) greater shock absorption properties under the forepart of the shoe are also associated with lower injury risk in recreational runners, (2) runners with low body mass experience a lower injury risk in shoes with greater shock absorption properties,^17^ and (3) greater perceived cushioning is related to lower injury risk.

## Methods

### Study design

**Figure 1:**
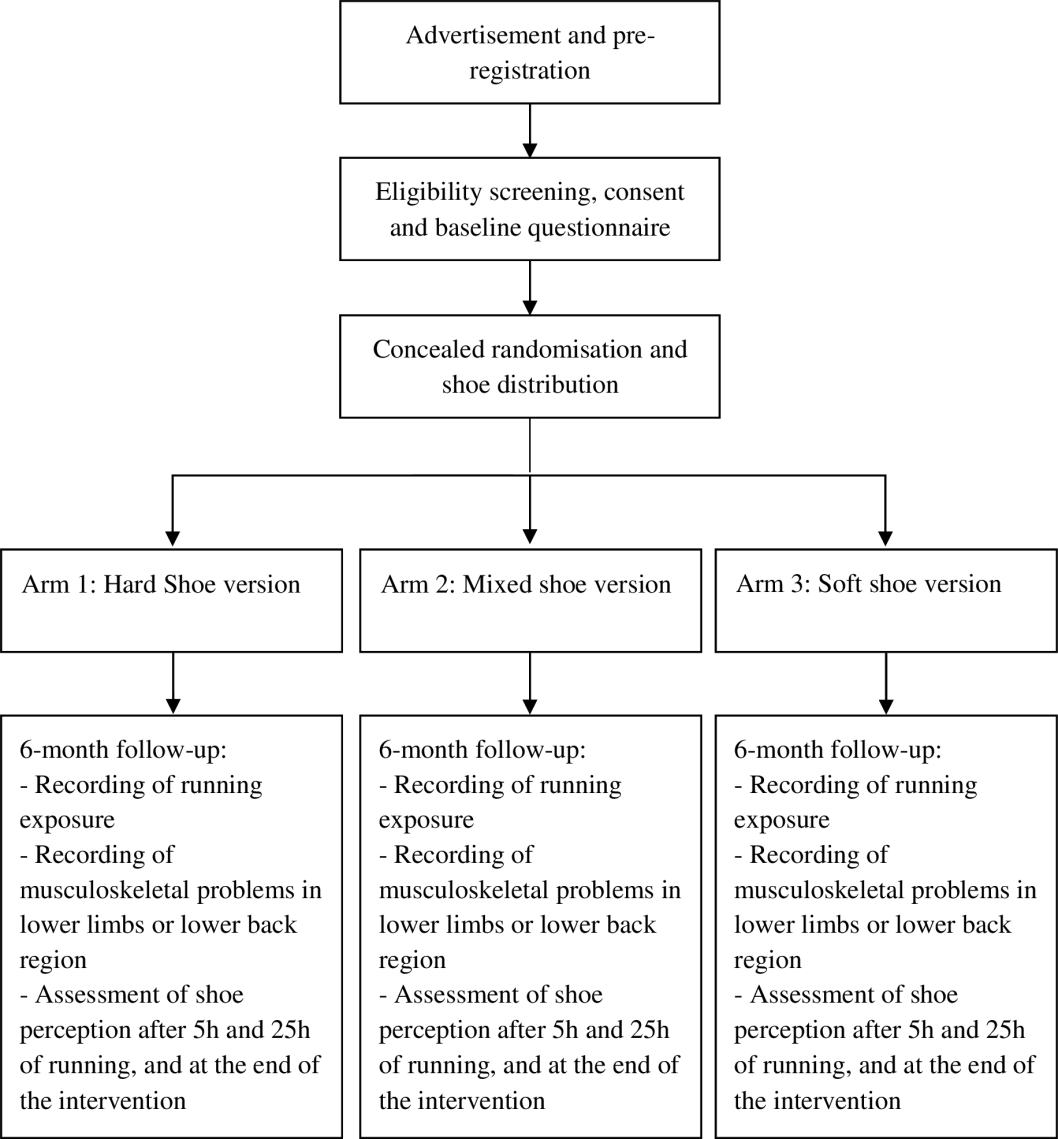
Trial design.

This study is a randomised trial with an intervention period of 6 months to compare three running shoe versions, which differ with respect to their cushioning properties and position (ie, rear and forepart of the shoe). A renowned sports equipment manufacturer provides the running shoe versions. The participants, as well as the assessors, will be blinded to group allocation. The design of the trial is illustrated in figure 1. The protocol conforms to the Recommendations for Interventional Trials (SPIRIT, (online supplemental material 1), has been registered on https://clinicaltrials.gov/ (NCT06384872, 02/08/2024) and was approved by the National Ethics Committee for Research (CNER; Ref: 202405/02 v2.0)). Written informed consent will be obtained from all participants (online supplemental material 2).

### Study population

Healthy leisure-time runners will be recruited through advertisements in local newspapers and press releases within Luxembourg and via social media from January 2025 to June 2025. Participants will be informed that they will randomly receive one of the three study shoe versions to be worn during the intervention period for all their running activities. Healthy volunteers will be considered eligible if they are aged between 18 and 65 years, willing to use the study shoes for each running session (and only for running activities), use a sports watch for training data recording, are Strava users (or are willing to create an account) and signed the electronic informed consent. Volunteers will be excluded in case of any contraindication to running training, including cardiovascular/respiratory, any disease or running impeding injury/condition at the time of initial inclusion, a history of surgery to the lower limbs or, the lower back within the previous 12 months, any known degenerative conditions, the use of orthopaedic insoles for physical activity, or any running injury (ie, a physical pain or complaint related to running practice that causes the runner to interrupt or modify his/her training for at least 1 week) over the month before study inclusion. No limitation will be set regarding running experience, running level (performance), body mass or Body Mass Index.

### Intervention

**Figure 2:**
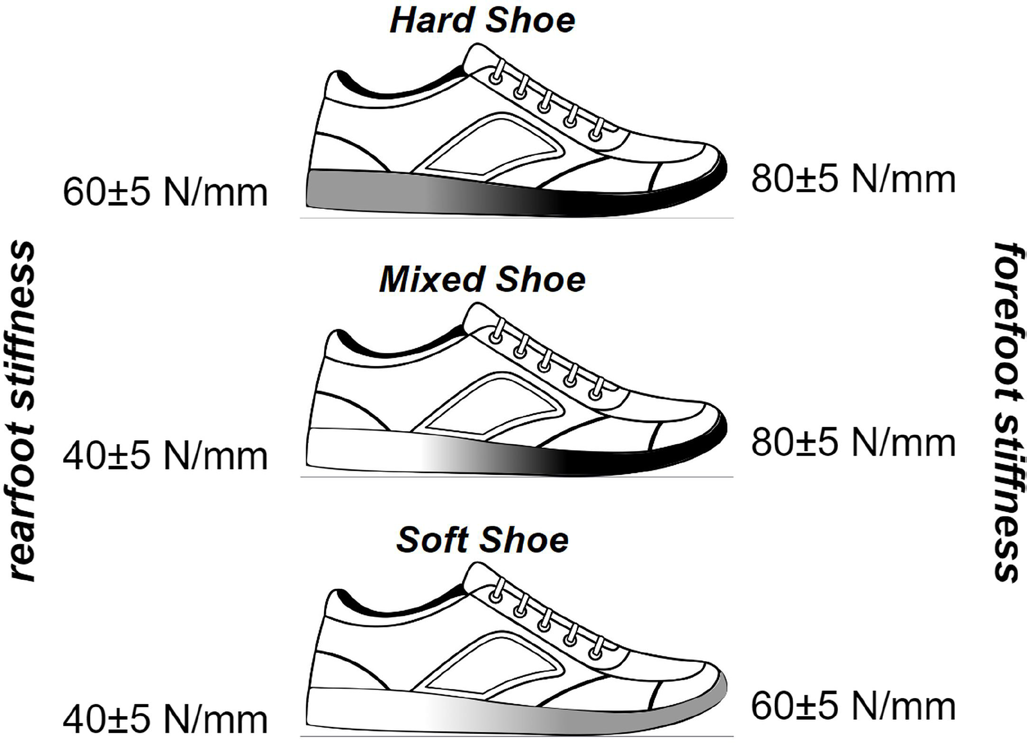
Study shoe versions.

The study shoes are prototypes derived from a model available on the market (Kiprun KS 500 2) and will be anonymised for the purpose of this trial. The soles of the shoes will be customised for the study. The three running shoe versions will have the same design, except for their cushioning properties at the rear and forepart of the shoe, which will differ by about 40% and 30%, respectively (figure 2). Nevertheless, cushioning properties will remain within the range of values of the shoes available on the market (linear equivalent stiffness: 40±5 to 60±5 and 60±5 to 80±5 N/mm, at the rear and forepart of the shoe, respectively). We expect a difference in shoe weight lower than 30 g between the three versions (for size 43). The difference in cushioning properties between shoe versions will be created by modifying the type of foam and the foaming process. A set of shoes (5 pairs per version, shoe size 43) will be tested for stiffness properties by the manufacturer according to a standardised protocol (Impact test: ASTM F1614, Procedure A)^26^ to provide accurate data on the technical specifications (stiffness and dissipated energy). The cushioning properties (linear equivalent stiffness) of the study shoe versions will be characterised as follows (see also figure 2): hard shoe version (‘Hard-Hard’—about 60±5 and 80±5 N/mm at the rear and forepart of the shoe, respectively), mixed shoe version (‘Soft-Hard’—about 40±5 and 80±5 N/mm) and soft shoe version (‘Soft-Soft’—about 40±5 and 60±5 N/mm). The main hypothesis will be tested by comparing the mixed shoe version with the hard shoe version, while the secondary hypothesis will be tested by comparing the soft shoe version with both the hard and mixed shoe versions.

### Stratified randomisation

Participants will be randomised at a 1:1:1 ratio to one of the three treatment arms. A block randomisation (block size 12) will be performed. Randomisation will be stratified by sex, as the latter influences body mass. Based on data from previous studies, the study population is expected to include about 40% of women. A statistician (at Luxembourg Institute of Health) who is not involved in any other part of the study will provide two pre-established randomisation lists before the beginning of recruitment. The participant identification number will comprise of four digits: the first digit corresponding to the participant’s sex (1=female, 2=male), followed by three digits corresponding to the randomisation number. The study groups and shoes will be coded, and an IT developer will upload the randomisation lists to the data collection system. Then, the data collection system will provide the investigator in charge of the recruitment with a study group number for each participant, according to the randomisation lists. The investigator will upload the shoe number according to the shoe size chosen and study arm so that the electronic system will perform cross-validation. The investigators in charge of the recruitment, the follow-up and data quality check, and the participants will be blinded regarding the shoe version distributed. The shoe code will be broken after the completion of data analysis.

### Data collection

Participants must fill in a baseline questionnaire to collect information regarding sociodemographic characteristics, running experience, training habits, running competitions performed and injury history. The intervention period will start as soon as the participants receive their study shoes and will last 6 months. During this intervention period, the participants are required to use the study shoes for all their running activities, to continue their usual training or follow their planned schedule and to fill out the weekly questionnaire on musculoskeletal problems, as well as on the use of the study shoes during the follow-up. Data on running practice will be downloaded from sports watches and their respective app via Strava in our dedicated electronic system. The system will collect the daily step count via Google Fit and the Apple Health mobile app. Information on using the study shoes will be collected via a weekly questionnaire. Data on cushioning perception will be collected three times using a dedicated questionnaire distributed when the runner has cumulated 5 hours and 25 hours of running and at the end of the intervention, respectively (online supplemental material 3).

Participants will be asked to complete a weekly questionnaire on any problem in the lower limbs or lower back region experienced during the previous 7 days (the Oslo Sports Trauma Research Centre (OSTRC) Overuse Injury Questionnaire).^27^ On top of the four basic questions, information regarding the following is required: date of symptom onset, sports discipline, anatomical location, type of injury, recurrence (ie, new, recurrent, exacerbation or subsequent injury), mode of onset and estimated return date to full participation (online supplemental material 4). Individual e-mail reminders will be sent to the participants who do not complete a weekly questionnaire. Personal phone calls will be made to participants who do not react to the e-mail reminders and if the reported information in the injury form is inconsistent. Study participation will be terminated, and the participant will be right-censored if he/she does not fill in the weekly injury form for two consecutive weeks, does not reply (or react) to the automatic reminders and cannot be reached by phone by the research team.

### Study outcomes

The primary outcome is the first running-related injury occurring during the follow-up and defined as any running-related physical complaint in the lower limbs or lower back region that causes a restriction on or stoppage of running (distance, speed, duration or training) for at least 7 days. Practically, this corresponds to injury forms where the participant answered options (c) or (d) in question 1 or options (b), (c) or (d) in question 2 or question 3 of the OSTRC-O questionnaire.^27^ Injury duration will be computed as the days between the date of injury or symptom onset and the date of return to full participation reported in our electronic system (via a dedicated form). Secondary outcomes are as follows: (1) first running-related physical complaint in the lower limbs or lower back region that causes a restriction on or stoppage of running (distance, speed, duration or training) for at least 1 day (ie, injury forms with options (c) or (d) in question 1 or options (b), (c) or (d) in question 2 or question 3 of the OSTRC-O questionnaire, without any minimal duration), (2) first physical complaint including pain, ache, joint instability, stiffness or any other complaint resulting from participating in running activities, including but irrespective of the need of medical attention or time-loss (ie, injury forms with option (b) in question 1 of the OSTRC questionnaire will also be considered), (3) first substantial running-related injury (ie, injury forms where the participant answered options (c) or (d) in question 2 or question 3 of the OSTRC-O questionnaire) and (4) first overuse running-related injury (ie, injury forms where the participant answered option (a) in question 10 will be excluded).

### Sample size

A sample size calculation for Cox regression was used to determine the number of participants needed for the study’s primary hypothesis. With an alpha of 0.05 and a power of 80%, an average injury rate of 30%,^28 29^ an expected HR=1.50 between groups, 33% of participants randomised to each shoe group and an expected drop-out rate of 10%, the total number of participants required is 1068 (ie, 356 participants per group).

### Statistics

Descriptive statistics will be presented as mean and SD for normally distributed continuous variables, median and range for skewed continuous variables, and count and percentage for categorical data, respectively. Shock absorption properties of the three shoe versions will be compared using an analysis of variance while considering the measurement uncertainty of the mechanical test.

Injury incidence will be computed for each outcome as the number of injuries per 1000 hours of running exposure. Injury prevalence will be computed for each outcome and per week by dividing the number of participants who reported an injury by the number of respondents of that week. Average weekly prevalence will be calculated separately for each outcome for the main anatomic locations.

To investigate the effect of shoe version, body mass and other possible risk factors on injury risk, subdistribution HR (sHR) with 95% CIs for the event of interest will be calculated using Fine-Gray models to account for competing risk. The date of inclusion and date of injury or censoring will be basic data used to calculate the time at risk, expressed in hours spent running and defined as the time scale.^14^ Participants will be right-censored if lost to follow-up or at the end of the intervention period. Average running practice characteristics will be computed for each participant over their specific period of observation. The assumption of proportional hazards will be evaluated using log-minus-log plots and Schoenfeld’s global test. First, unadjusted sHR will be estimated for shoe versions, body mass and other potential risk factors, such as running training characteristics. Subsequently, the variables with a p value <0.200 and well-established potential confounders will be included in an adjusted model to determine whether the model is improved with further adjustment. The model that best fits will be determined based on the log-likelihoods. Stratified analyses will be performed to investigate if the effect of shoe cushioning on injury risk is modified by sex, body mass (using the median value of body mass as cut-off, independently in men and women) or Body Mass Index. Finally, mediation analysis will investigate if the effect of cushioning on injury risk is partially explained by cushioning perception (and comfort).

Sensitivity analyses will first investigate the potential impact of the transition to the study shoe on the findings by excluding the participants who sustained an injury over the first 2 weeks (ie, left-truncated models). Second, depending on the participants’ compliance to the intervention (ie, use of the study shoes for each running session), we will exclude the participants who did not use the study shoes for (eg,) a minimum of 50% of their running sessions.

Exploratory analyses will investigate whether weight-bearing locomotion activities (ie, number of steps per day besides running practice) represent an independent risk factor for running-related injury, as well as whether injury risk depends on the socioeconomical status of the runners (eg, level of education, socioprofessional category, gross annual household income). All analyses will be performed using STATA/SE V.15.

## Discussion

Previous work has provided evidence that shoe cushioning effectively reduces injury risk in leisure-time runners.^17^ However, some important knowledge gaps remain to fill before designing the most effective running shoe for injury prevention. First, while the market is rapidly evolving towards greater cushioning, notably through the advent of super-critical foam technology since 2018, there is currently no data on the effect of these new materials (with greater cushioning properties) on injury risk. Second, running shoe design has evolved over the last decades, and runners are increasingly interested in shoe models with large stack height (and, consequently, greater cushioning) at the forepart of the shoe. While most literature has focused on cushioning (stiffness) at the rear part of the shoe to attenuate impact forces, force components unrelated to heel impact account for about half of the impact peak magnitude,^30^ which calls for more research on the possible effect of cushioning at the forepart of the shoe on injury risk.

### Limitations

This study will be a participants’ and assessors’ blinded randomised trial with a 6-month intervention on 1000+ leisure-time runners, with objective measurement of running exposure via the participants’ sports watches. However, the participants will self-report musculoskeletal problems in the lower limbs and lower back region, which may limit the accuracy of data related to injury type. Furthermore, the study may suffer from low statistical power when investigating the risk of developing specific injury types. Regarding the study population, more competitive runners might be under-represented in the study because of a lower readiness to comply with the study requirements (ie, use of the study shoes for all running sessions). Lastly, no biomechanical analysis will be performed to investigate the mechanisms responsible for the potential effects of greater cushioning.

### Dissemination

Study results will be disseminated via publications in peer-reviewed scientific journals and presentations at international conferences. We also aim to disseminate our results through popular specialised magazines, websites and webinars.

## supplementary material

10.1136/bmjsem-2024-002217 online supplemental file 1

10.1136/bmjsem-2024-002217 online supplemental file 2

10.1136/bmjsem-2024-002217 online supplemental file 3

10.1136/bmjsem-2024-002217 online supplemental file 4

## Data Availability

No data are available.
