# Supplementary material for: Running shoe cushioning properties at the rearfoot and forefoot and their relationship to injury: study protocol for a randomised controlled trial on leisure-time runners
Source: BMJ Open Sport Exerc Med. 2024 Oct 11;10(4):e002217. doi: 10.1136/bmjsem-2024-002217 (PMC11481106; doi:10.1136/bmjsem-2024-002217)
Supplement: online supplemental file 2 [file bmjsem-10-4-s002.pdf]

Information for the participant and Informed consent form

Title of the study: Effect of foam properties and cushioning position of running shoes on injury risk in leisure-time runners: A randomised trial

Number of the study, acronym: RRI_Interv5

Sponsors of the study: Luxembourg Institute of Health and Decathlon

Scientific Principal Investigator of the study: xxx

Medical Principal Investigator of the study:

Name: xxx

Contact details: xxx

Telephone: xxx

Research team: (+352 26970xxx) [xxx@lih.lu]

1 INTRODUCTION

You are invited to take part in a study. The purpose of this document is to provide you with some information on the study to help you decide whether or not you would like to take part. Your participation is entirely voluntary. If you decide to take part, you may withdraw at any time without having to give any reason. Similarly, should your doctor deem your state of health unsuitable at any point, he/she will ask you to withdraw from the study. This study was authorized by the Ministry of Health on XX/XX/XX and has received a favourable opinion from the National Research Ethics Committee. However, you should not take this latter information as an incentive to participate in this study. This study is subject to the Grand Ducal Regulation 8 March 2018.

2 WHAT IS THE PURPOSE OF THE STUDY PROGRAMME?

It is important that you understand why the research is being done and what it will involve. Please read the following information carefully. Please ask the researcher if there is anything that is not clear or if you need more information.

The purpose of this study is to investigate the effect of different cushioning solutions on injury risk in running. The project focuses on the influence of cushioning material at both the rear and the forepart of running shoes on injuries in leisure-time runners.

Our previous work has shown that shoe cushioning is effective in reducing injury risk in leisure-time runners. However, the market is still evolving towards greater cushioning, notably through the advent of new foam technology, and the effect of these new materials (with greater cushioning properties) on injury risk is unknown. Furthermore, there is also increasing interest from runners in shoe models with large stack height (and greater cushioning) at the forepart of the shoe, while the specific effect of cushioning at the forepart of the shoe on injury risk has never been investigated.

3 HOW WILL THE STUDY BE CONDUCTED?

If you are interested in this study, we will ask you to visit our dedicate electronic platform that was specifically designed for the study to check the eligibility criteria. If you believe that you meet the inclusion criteria and are willing to participate in the study, you will be asked to:

- Create an account in our dedicated electronic system.
- Read the documentation about the study.
- Provide your consent to participate in the study.
- Fill in a baseline questionnaire.
- Finalize the registration by setting an appointment with the research team via the electronic system.

During the initial visit at LIH, your eligibility to the study will be confirmed. You will also have the possibility to ask further questions about the study. If eligible, you will be allocated to one of the study groups at random, and receive the study shoes, as well as some explanation on the data collection system. There will be three shoe conditions, with cushioning properties similar to those from running shoes currently available in the market:

- Model “Hard-Hard” at the rear and forepart of the shoe, respectively.
- Model “Soft-Hard” at the rear and forepart of the shoe, respectively.
- Model “Soft-Soft” at the rear and forepart of the shoe, respectively.

During the 6-month follow-up period, data on running practice will be downloaded from your sports watch (e.g., Garmin, Polar and Suunto) and the respective application (e.g., Garmin Connect) via Strava. You will be asked to:

- Use the study shoes for each running training session, and only for running activities;
- Confirm (or not) the use of the study shoes for each running session on a weekly basis;
- Report any physical complaints on a weekly basis;
- Fill out a questionnaire on the perception of cushioning at three time points (after 5h of running, after 25h of running, and at the end of follow-up);
- Pursue your usual or planned training programme. No instruction on running training will be provided.
- Return the shoes at the end of the study to analyse any cushioning degradation.

<u>Below is a summary of all the tasks you'll be asked to complete when taking part in the study:</u>

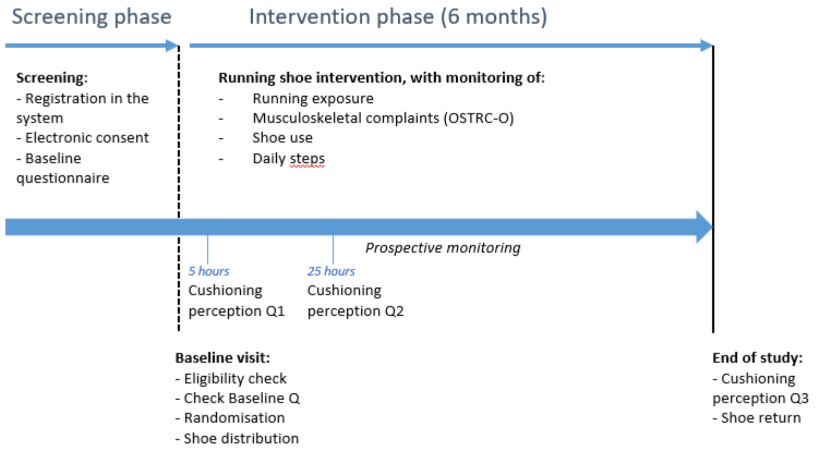

4 WHAT WILL HAPPEN TO MY DATA?

You are invited to give data for this study (primary use).

At the beginning of the study, you will be asked to fill in a baseline questionnaire that aims to collect information regarding your socio-demographics, your health (weight, height, chronic diseases, previous running-related injury), as well as your running experience, training habits, competition and performance in running.

During the study:

- Data on your running practice will be downloaded from your sports watch (e.g., Garmin, Polar, Suunto) and their respective applications via Strava.
- Information on the use of the study shoes will be collected via a validation dashboard on a weekly basis.
- You will be asked to fill in a weekly questionnaire on any physical complaints experienced during the past 7 days (the Oslo Sports Trauma Research Centre (OSTRC) Overuse Injury Questionnaire).

Finally, a questionnaire on the perception of cushioning will be sent at three time points during the study: after 5h of running, after 25h of running, and at the end of follow-up.

All the data collected will be entered in a secured server in a pseudonymised form, which means that your name will be replaced by a confidential reference code. Only the member of the research team responsible for the follow-up of the participants will have access to your contact details in case some information should be verified/confirmed.

Consult the Data Protection Notice in order to have more details on these data and to be informed about the processing your data and your rights.

5 WHAT ARE THE POSSIBLE RISKS AND SIDE EFFECTS?

By taking part in this clinical study, you acknowledge that you understand that some procedures performed may pose certain risks, also when minimal, for your health and well-being.

The risk of traumatic injuries linked to the practice of physical activity is present but negligible. There is no risk previously documented, or anticipated adverse effect, with the use of the new cushioning materials such as the one that will be used in the study shoe versions.

There are also risks associated with the fact that the study involves the recording of data online (e.g. hacking, which endangers the confidentiality of participants' personal data). This risk is low but exists. LIH has implemented extensive data protection measures to minimize this risk. These measures are explained in the section "Confidentiality and Protection of Personal Data".

6 WHAT ARE THE BENEFITS OF TAKING PART IN THE STUDY?

You may not benefit directly by taking part in this study. Your participation is voluntary. You will receive no form of compensation for your involvement or for any subsequent developments resulting from the study.

While the immediate benefits may be limited for most of the study participants, the study outcomes will help researchers better understand the relationship between shoe cushioning and injury risk, and the industry to make important decisions for the design of their future running shoes.

7 SECONDARY USE OF MY DATA

If you agree to participate in this study, you will also be asked to authorize the further use (secondary use) of your data (with the exception of your direct identifying data) for future research in the field of running-related injury.

For more information, please read the information sheet on the secondary use of your data in future research. If you wish to authorize the secondary use of your data, please complete and sign the specific consent form for these uses.

8 CONFIDENTIALITY AND PROTECTION OF PERSONAL DATA

Your data will be treated as strictly confidential. It will also be pseudonymised, meaning that your name will be replaced by a confidential reference code. This code alone will not directly identify you and will only be used to process your data for scientific purposes. Your identity will never be disclosed in any document produced for the public or for other institutions. Apart from the Medical Principal Investigator, only a limited number of authorized persons from the research team at LIH will have access to your identity in a confidential and secure manner.

Your personal data will be needed during the study. In accordance with the applicable legal provisions, your personal data and the correspondence table will be stored for a period of 2 years following the last visit of the last patient participating in the study (expected in September 2025). At the end of these 2 years, the nominative data will be destroyed.

Consult the Data Protection Notice in order to obtain detailed information in regards to the processing of your personal data and your rights, in accordance with the principles of lawfulness, fairness and transparency.

9 COSTS ASSOCIATED WITH YOUR PARTICIPATION

If you decide to take part, neither you nor your insurance company will be entitled to any form of remuneration.

10 INSURANCE

Any involvement in a clinical study carries a risk, however small. If you become ill, experience any undesirable side effects or suffer any injuries as a result of the study, you should talk to your doctor. Your doctor will ensure that you receive the appropriate medical care and advice both during and after the study. The insurance policy taken out by Luxembourg Institute of Health (Zurich Insurance plc, Belgium Branch, Da Vincilaan 5, B-1930 Zaventem) will cover the cost of any damage that may result from the study.

11 YOUR DECISION TO TAKE PART

If you choose to take part, you may withdraw at any time without giving a reason.

Before you take part in the study, you will need to provide your written consent by completing the form below. The research team will keep a copy of this document in a safe place. You will receive your own copy of the document. Should you have any questions about this study, you can contact the research team at any time.

If you decide to take part in this clinical study, we ask that you:

- give your full cooperation throughout the study;
- do not conceal any information on the state of your health or any symptoms that you might experience;
- do not take part in any other clinical research project on an experimental treatment, whether it involves a drug, a medical device or a procedure, while you are taking part in this study.

If you would like further information on the study, please contact running@lih.lu.

Consent form

- I declare that I have read and understood the information provided above.
- I have had enough time to consider my involvement in the study and to discuss it with a person of my choice, such as a member of my family. I have had the opportunity to ask all of the questions that have occurred to me in relation to the study and I have received satisfactory responses to each of them.
- I am aware of what is expected of me as a participant in this study.
- I am aware that my participation in this study is entirely voluntary and that I am free to withdraw at any time without giving a reason for my decision and without being held liable for any material or non-material damages. I will only need to inform the research team of my decision.
- I accept that the results from this study may be disclosed and reported in scientific publications. The way in which these results are presented will in no way enable me to be identified, either directly or indirectly.
- As described in the information document on the processing of my personal data as part of this study, I understand that any personal information gathered in relation to this study will be treated as strictly confidential, in accordance with the General Data Protection Regulation (EU) 2016/679 of 27 April 2016 (known as the GDPR) and all subsequent texts replacing or supplementing this Regulation (in particular, Luxembourg's law of 1 August 2018 on the organisation of its National Commission for Data Protection and the implementation of the GDPR).
- I have received a copy of the present document, together with the information document explaining how my personal data will be processed as part of this study, and of the specific information document on the secondary use of my data.

In the table below, if you answer NO to any of the statements followed by an asterisk (*), you will not be able to take part in the study, as these are essential to its objectives.

Consent Form		
1. I willing to agree to take part in this study under the terms set out on the above information sheet (*).	NO	☐
	YES	☐
2. I agree to being contacted again by the Scientific Principal Investigator with a view to taking part in another study.	NO	☐
	YES	☐
3. I hereby give consent to the processing of my personal data as part of the study based on the terms and conditions set out in the information document explaining how my personal data will be processed (*).	NO	☐
	YES	☐

Participant's first and last name(s): ....

Date of signature (day/month/year): ....

Signature of participant: ....

One copy for the participant and one copy to the institution responsible for the study.
