# Supplementary material for: Running shoe cushioning properties at the rearfoot and forefoot and their relationship to injury: study protocol for a randomised controlled trial on leisure-time runners
Source: BMJ Open Sport Exerc Med. 2024 Oct 11;10(4):e002217. doi: 10.1136/bmjsem-2024-002217 (PMC11481106; doi:10.1136/bmjsem-2024-002217)
Supplement: online supplemental file 3 [file bmjsem-10-4-s003.pdf]

1. How do you **rate** the **overall cushioning** of the sole of your shoe?

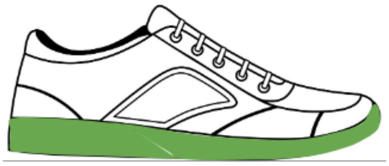

0	1	2	3	4	5	6	7	8	9	10
☐	☐	☐	☐	☐	☐	☐	☐	☐	☐	☐

I do not like it at all — I really like it

Heel area

2. How do you **perceive the intensity** of the **cushioning (insertion) of the heel** in the sole of your shoe?

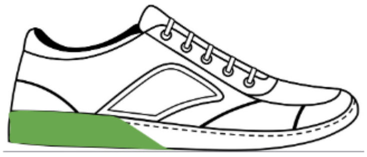

0	1	2	3	4	5	6	7	8	9	10
☐	☐	☐	☐	☐	☐	☐	☐	☐	☐	☐

No cushioning at all — Maximum cushioning

3. In terms of **heel cushioning**, do you think this shoe is **suitable** for running?

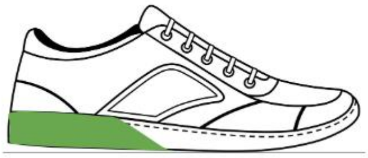

☐ Not at all suitable

☐ Rather unsuitable

☐ Rather suitable

☐ Fully suitable

Forefoot area

4. How do you **perceive the intensity** of the **cushioning (insertion) of the forefoot** into the sole of your shoe?

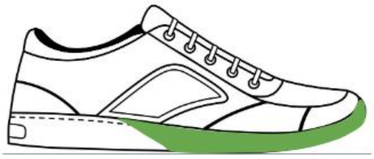

0	1	2	3	4	5	6	7	8	9	10
☐	☐	☐	☐	☐	☐	☐	☐	☐	☐	☐

No cushioning at all — Maximum cushioning

5. In terms of **forefoot cushioning**, do you think this shoe is **suitable** for running?

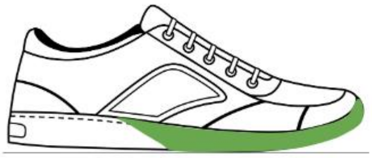

☐	☐	☐	☐
Not at all suitable	Rather unsuitable	Rather suitable	Fully suitable
