# Supplementary material for: Running shoe cushioning properties at the rearfoot and forefoot and their relationship to injury: study protocol for a randomised controlled trial on leisure-time runners
Source: BMJ Open Sport Exerc Med. 2024 Oct 11;10(4):e002217. doi: 10.1136/bmjsem-2024-002217 (PMC11481106; doi:10.1136/bmjsem-2024-002217)
Supplement: online supplemental file 4 [file bmjsem-10-4-s004.pdf]

Injury Data Collection Form

Please answer all questions regardless of whether or not you have problems in your lower limbs or lower back region. Select the alternative that is most appropriate for you, and in the case that you are unsure, try to answer as best you can anyway.

The term ‘lower limbs or lower back problems’ refers to any pain, ache, stiffness, clicking/catching, swelling, instability/giving way, locking or other complaints related to your lower limbs or lower back.

Question #1—Participation

Have you had any difficulties participating in training and competition due to lower limbs or lower back problems during the past 7 days?

a. Full participation without lower limbs or lower back problems
b. Full participation, but with a lower limbs or lower back problems
c. Reduced participation due to a lower limbs or lower back problems
d. Could not participate due to a lower limbs or lower back problems
   - ➔ *Questionnaire completed if option “A” (Question 1, 2 and 3 should be “A”)*
   - ➔ *Jump to question 4 if option “D” (Question 2 and 3 should be “D”)*

Question #2—Modified training/competition

To what extent have you modified your training or competition due to lower limbs or lower back problems during the past 7 days?

a. No modification
b. To a minor extend
c. To a moderate extend
d. To a major extend

Question #3—Performance

To what extent have lower limbs or lower back problems affected your performance during the past 7 days?

a. No effect
b. To a minor extend
c. To a moderate extend
d. To a major extend

Question #4—Symptoms

To what extent have you experienced lower limbs or lower back pain related to running during the past 7 days?

a. No pain
b. Mild pain
c. Moderate pain
d. Severe pain

Question #5 – Injury date

What is the date of injury or symptom onset?

- [Date]

Question #6 – Sports discipline

Which sport did you practice when the first symptom occurred?

- [Predefined list of sports]
- Injury not related to sport

Question #7 – Anatomical location

What is the anatomical location of the main complaint?

- Foot
- Ankle
- Lower leg
- Knee
- Thigh
- Hip/groin
- Buttock/pelvis
- Lower back region

Question #8 – Type of injury

What is the main tissue concerned?

- Tendon
- Muscle
- Capsule and Ligament
- Bone structure
- Other joint structure
- Other overuse injury/undefined
- I do not know

Question #9 – Index or Subsequent injury

Is it your first injury recorded (or experienced over that last 6 months)?

a. Yes
b. No, it is a recurrent injury to the same location and tissue as a previous injury
c. No it is an exacerbation of a previous injury that was not fully healed
d. No, it is a subsequent injury that affects another site or other tissues from the same anatomical location

Question #10 – Mode of onset

What is the mechanism of injury?

a. Acute mechanism with sudden onset (e.g., ankle sprain)
b. Repetitive mechanism with sudden onset (e.g., muscle tears at the end of a running session)
c. Repetitive mechanism with gradual onset (e.g., Achilles tendinopathy)

Question #11 – Estimated return date

What is the estimated return date to full participation to running activities without any restriction?

- [Date]
